# Differential vulnerability of locus coeruleus and dorsal raphe neurons to chronic methamphetamine-induced degeneration

**DOI:** 10.3389/fncel.2022.949923

**Published:** 2022-07-22

**Authors:** Yijuan Du, Sanghoon Choi, Alexander Pilski, Steven M. Graves

**Affiliations:** Department of Pharmacology, University of Minnesota, Minneapolis, MN, United States

**Keywords:** methamphetamine, monoamine oxidase, mitochondria, oxidant stress, L-type Ca^2+^ channel, neurodegeneration, locus coeruleus, dorsal raphe

## Abstract

Methamphetamine (meth) increases monoamine oxidase (MAO)-dependent mitochondrial stress in axons of substantia nigra pars compacta (SNc), and ventral tegmental area (VTA) dopamine neurons. Chronic administration of meth results in SNc degeneration and MAO inhibition is neuroprotective, whereas, the VTA is resistant to degeneration. This differential vulnerability is attributed, at least in part, to the presence of L-type Ca^2+^ channel-dependent mitochondrial stress in SNc but not VTA dopamine neurons. MAO is also expressed in other monoaminergic neurons such as noradrenergic locus coeruleus (LC) and serotonergic dorsal raphe (DR) neurons. The impact of meth on mitochondrial stress in LC and DR neurons is unknown. In the current study we used a genetically encoded redox biosensor to investigate meth-induced MAO-dependent mitochondrial stress in LC and DR neurons. Similar to SNc and VTA neurons, meth increased MAO-dependent mitochondrial stress in axonal but not somatic compartments of LC norepinephrine and DR serotonin neurons. Chronic meth administration (5 mg/kg; 28-day) resulted in degeneration of LC neurons and MAO inhibition was neuroprotective whereas DR neurons were resistant to degeneration. Activating L-type Ca^2+^ channels increased mitochondrial stress in LC but not DR axons and inhibiting L-type Ca^2+^ channels *in vivo* with isradipine prevented meth-induced LC degeneration. These data suggest that similar to recent findings in SNc and VTA dopamine neurons, the differential vulnerability between LC and DR neurons can be attributed to the presence of L-type Ca^2+^ channel-dependent mitochondrial stress. Taken together, the present study demonstrates that both meth-induced MAO- and L-type Ca^2+^ channel-dependent mitochondrial stress are necessary for chronic meth-induced neurodegeneration.

## Introduction

Methamphetamine (meth) is an addictive and especially dangerous psychostimulant that is capable of producing neurotoxicity. Per the 2019 National Survey on Drug Use and Health (NSDUH) approximately two million people > 12 years of age reported using meth and approximately one million reported a meth use disorder. In addition to abuse liability, meth is neurotoxic, particularly to the nigrostriatal system. In human subjects, meth abuse decreases levels of striatal dopamine content, dopamine transporters (DAT), and tyrosine hydroxylases (TH) (Wilson et al., 1996; Mccann et al., 1998; Sekine et al., 2001; Volkow et al., 2001; Moszczynska et al., 2004). Consistent with deleterious effects on the nigrostriatal system, patients with a history of meth abuse have an increased risk for developing Parkinson’s disease (Callaghan et al., 2010, 2012; Curtin et al., 2015), the most common neurodegenerative movement disorder in which nigrostriatal degeneration is a hallmark. Recent work has linked the pharmacological actions of meth to mitochondrial oxidant stress and nigrostriatal degeneration (Graves et al., 2020, 2021; Du et al., 2021).

Meth binds to and disrupts vesicular monoamine transporter 2 (VMAT2) function resulting in increased concentrations of cytosolic dopamine (Sulzer et al., 2005; Freyberg et al., 2016). One outcome of elevating cytosolic dopamine is mitochondrial oxidant stress due to monoamine oxidase (MAO) metabolism. The deamination of cytosolic dopamine by MAO enzymes, which are tethered to mitochondria, generates electrons. These electrons enter the mitochondrial intermembrane space, increasing mitochondrial oxidant stress (Graves et al., 2020). Persistently engaging this MAO-dependent mitochondrial stress pathway *via* chronic 28-day *in vivo* administration of meth (5 mg/kg) results in overt degeneration of substantia nigra pars compacta (SNc) dopamine neurons whereas inhibiting MAO prevents degeneration (Du et al., 2021; Graves et al., 2021). Administration of meth using an acute binge paradigm also produces SNc degeneration (Sonsalla et al., 1996; Ares-Santos et al., 2014; Mendieta et al., 2016). However, neighboring ventral tegmental area (VTA) dopamine neurons are resistant to chronic meth-induced degeneration despite also having meth-induced MAO-dependent mitochondrial stress (Du et al., 2021). A differentiating feature between SNc and VTA dopamine neurons is the presence of L-type Ca^2+^-dependent mitochondrial oxidant stress in SNc but not VTA neurons (Chan et al., 2007; Guzman et al., 2010; Surmeier et al., 2017; Du et al., 2021). Like MAO inhibition, L-type Ca^2+^ channel inhibition similarly prevents chronic meth-induced SNc degeneration, indicating that meth drives degeneration through MAO-dependent mitochondrial stress whereas vulnerability is determined, at least in part, by the presence of L-type Ca^2+^-dependent mitochondrial stress (Du et al., 2021).

In addition to packaging dopamine into vesicles, VMAT2 is also used to package norepinephrine and serotonin, making these neurotransmitter systems a target for meth to increase cytosolic neurotransmitter concentrations. Like with dopamine, MAO enzymes also metabolize both norepinephrine and serotonin; norepinephrine neurons in the locus coeruleus (LC) and serotonin neurons in the dorsal raphe (DR) were therefore hypothesized to have similar meth-induced MAO-dependent mitochondrial oxidant stress. LC norepinephrine neurons, like SNc dopamine neurons, exhibit somatic L-type Ca^2+^ channel-dependent mitochondrial stress (Sanchez-Padilla et al., 2014). However, meth-induced MAO-dependent mitochondrial stress in dopamine neurons occurs in axonal but not somatic compartments (Graves et al., 2020; Du et al., 2021) and it is unknown whether LC neurons, like SNc neurons (Du et al., 2021), also have L-type Ca^2+^ channel-dependent mitochondrial oxidant stress in the axonal compartment. The status of L-type Ca^2+^ channel-dependent mitochondrial oxidant stress in serotonergic DR neurons is unknown. Whether LC and DR neurons are vulnerable to chronic meth-induced degeneration and the potential involvement of MAO enzymes and L-type Ca^2+^ channels remains to be explored.

The present study employed two-photon laser scanning microscopy in live *ex vivo* mouse brain slices to measure mitochondrial oxidant stress using a genetically encoded biosensor. Degeneration was quantified using unbiased stereology after chronic *in vivo* administration of meth for 28 consecutive days. We demonstrate that meth increased MAO-dependent mitochondrial oxidant stress in both LC and DR axons, whereas LC but not DR axons had L-type Ca^2+^ channel-dependent mitochondrial stress. Chronic meth administration resulted in LC but not DR degeneration with both MAO and L-type Ca^2+^ channel inhibition being neuroprotective. When combined with prior studies (Du et al., 2021; Graves et al., 2021), the current findings show that SNc and LC neurons are vulnerable to chronic meth-induced degeneration whereas VTA and DR neurons are resistant. Both SNc and LC degeneration is driven by meth-induced MAO-dependent mitochondrial stress while L-type Ca^2+^ channel-dependent mitochondrial stress differentiates vulnerable from resistant neuronal populations.

## Materials and methods

### Animals

All procedures were in accordance with the National Institutes of Health Guide for the Care and Use of Laboratory Animals and approved by the University of Minnesota Institutional Animal Care and Use Committee. C57Bl/6 mice and mice expressing Cre recombinase under the tyrosine hydroxylase (TH-Cre) or serotonin transporter reporters (SERT-Cre) on a C57Bl/6 background were group-housed with food and water provided *ad libitum* on a 12-h light/dark cycle with all *in vivo* treatments and experiments conducted during the light cycle; a total of 100 mice were used in the current report. Experiments were restricted to male subjects, because female subjects are more resistant to the deleterious effect of meth (Liu and Dluzen, 2006; Bourque et al., 2011a,b). All subjects were bred in-house and transgenic mice used in experiments were hemizygous for transgenes.

### Stereotaxic surgery

To examine mitochondrial oxidant stress, adeno-associated virus expressing the redox biosensor roGFP targeting the mitochondrial matrix (Sabharwal et al., 2013) under double-floxed inverse orientation (AAV9-DIO-mito-roGFP) was generated by the University of Minnesota Viral Vector and Cloning Core. Mice between 6 and 8 weeks of age were anesthetized using isoflurane and placed in a stereotaxic frame (Kopf Instruments) with a Cunningham adaptor (Harvard Apparatus). Anesthesia was maintained at a flow rate of 1.5–2.5% and anesthetic depth was monitored throughout the surgery. Meloxicam (2 mg/kg) was administered subcutaneously after which an incision was made to expose the skull. A small hole was drilled with a micro drill and 400 nL AAV9-DIO-mito-roGFP was injected into the locus coeruleus (LC, coordinates: AP: −5.45 mm, ML: 1.25 mm, and DV: 3.65 mm from bregma) in TH-Cre mice and into the dorsal raphe (DR, coordinates: AP: −4.5 mm, ML: 0 mm, and DV: 3 mm from bregma) in SERT-Cre mice using a thin glass pipette pulled on a P-1000 micropipette puller (Sutter Instruments). Mice received 2 mg/kg meloxicam subcutaneously for 3 days post-op and were allowed at least 10 days of recovery before being euthanized and *ex vivo* brain slices generated to examine mitochondrial oxidant stress.

### *Ex vivo* brain slice preparation and two-photon laser scanning microscopy

Live *ex vivo* brain slices entailing the LC or DR were collected to examine somatic mitochondrial oxidant stress and primary motor cortex (M1), which is innervated by both LC and DR axons, to examine axonal mitochondrial oxidant stress. Mice were anesthetized by intraperitoneal (i.p.) injection of ketamine (50 mg/kg)/xylazine (4.5 mg/kg), and transcardially perfused with ice-cold low Ca^2+^/high sucrose artificial cerebrospinal fluid (aCSF) containing (in mM) 49.0 NaCl, 2.5 KCl, 2.0 CaCl_2_, 10.0 MgCl_2_, 25.0 NaHCO_3_, 1.43 NaH_2_PO_4_, 2.5 glucose, and 5.0 sucrose. Coronal slices (220 μm thick) containing the LC, DR, or M1 were collected using a vibratome (Leica VT1200S). Slices were placed in a holding chamber containing normal aCSF (in mM): 124.0 NaCl, 3.0 KCl, 2.0 CaCl_2_, 1.0 MgCl_2_, 26.0 NaHCO_3_, 1.0 NaH_2_PO_4_, and 16.66 glucose (pH 7.4, 310–320 mOsm) for at least 30 min prior to imaging experiments; 95% O_2_/5% CO_2_ was continuously bubbled throughout. Mitochondrial oxidant stress was measured by imaging the redox-sensitive roGFP probe targeted to the mitochondrial matrix, as previously described (Graves et al., 2020, 2021; Du et al., 2021). Slices were transferred to a recording chamber continuously perfused with oxygenated aCSF at 32–34°C and fluorescence was measured using an Ultima Laser Scanning Microscope system (Bruker) with a Nikon FN-1 microscope, a 60X/1.00 NA lens, and PrairieView software. A two-photon laser (Chameleon Ultra II, Coherent Inc., Santa Clara, CA, United States) was used to excite roGFP with a 920 nm wavelength. Time-series images consisting of 60 frames were acquired at 0.195 μm × 0.195 μm pixels collected over ∼20 s with a 10–12 μs dwell time. After obtaining the test measurement the dynamic range of the probe was determined by sequentially perfusing 2 mM dithiothreitol, a reducing agent, and 200 μM aldrithiol, an oxidizing agent, and taking time-series images after each perfusion. Time-series images were analyzed offline, and fluorescence was measured in multiple regions of interest (ROIs) within the soma of LC noradrenergic and DR serotonergic neurons to determine somatic mitochondrial oxidant stress; axonal mitochondrial oxidant stress was similarly measured using multiple ROIs within the M1 motor cortex. Background fluorescence was subtracted and test measurements calculated as relative oxidation (Graves et al., 2020, 2021; Du et al., 2021). Rasagiline (1 μM) was used to inhibit MAO enzymes and Bay K8644 (10 μM) was used to activate L-type Ca^2+^ channels. For experiments with rasagiline, slices were preincubated in the holding chamber and bath perfused with meth + rasagiline consistent with prior studies (Graves et al., 2020, 2021; Du et al., 2021).

### *In vivo* drug administration

Male C57/Bl6 mice underwent a chronic 28-day treatment schedule with once-daily injections beginning at ∼8 weeks of age. Subjects were administered saline (10 ml/kg), methamphetamine hydrochloride (meth, 5 mg/kg, i.p.), or meth with the MAO inhibitor rasagiline (1 mg/kg, i.p.) administered as a 30-min pretreatment prior to each meth injection. Doses used and duration of administration were based on prior studies demonstrating that chronic 28-day administration of meth (5 mg/kg, i.p.) produces degeneration of SNc but not VTA neurons and that pretreatment MAO inhibition is neuroprotective (Du et al., 2021; Graves et al., 2021). The MAO inhibitor rasagiline is a MAO-B inhibitor and was chosen for the current investigation because meth-induced mitochondrial oxidant stress in SNc axons is similarly attenuated by both rasagiline and the MAO-A inhibitor clorgyline (Graves et al., 2020), rasagiline prevents chronic meth-induced SNc degeneration (Du et al., 2021; Graves et al., 2021), and rasagiline is clinically available, well-tolerated, and FDA-approved to treat symptoms in Parkinson’s disease. To study the effect of L-type Ca^2+^ channels, the clinically available negative allosteric modulator isradipine was dissolved in 50% DMSO/50% PEG300 at a concentration to achieve a dose of 3 mg/kg/day delivered *via* Alzet osmotic minipumps (model 2004) (Du et al., 2021; Graves et al., 2021). Minipumps were implanted subcutaneously under isoflurane anesthesia, following the manufacturer’s guidelines, 2 days before the first meth injection. Mice were sacrificed within 12 h of the last saline/meth injection.

### Immunohistochemistry

Mice were anesthetized by i.p. injection of ketamine (50 mg/kg)/xylazine (4.5 mg/kg) and transcardially perfused with 4% paraformaldehyde in PBS. Brains were post-fixed overnight and cryoprotected in 30% sucrose in PBS. Sections (40 μm) were sliced using a microtome (Leica SM2010R). Every third section throughout LC and DR was stained, a total of 7–8 slices for LC and 8 for DR. Every sixth section throughout the anterior portion of the primary motor cortex (M1, between 1.2 and 0.4 μm anterior from bregma) was stained for LC and DR axons, a total of four slices for each. Sections were treated with 20% formic acid for antigen retrieval and blocked with 5% normal donkey serum. Sections containing LC were stained for tyrosine hydroxylase (TH, primary antibody: rabbit anti-TH polyclonal AB152, Millipore, 1:2,000; secondary antibody: Alexa 555 donkey anti-rabbit A31572, Invitrogen, 1:200), and NeuN [primary antibody: mouse anti-NeuN monoclonal (1B7) ab104224, Abcam, 1:500; secondary antibody: Alexa 488 donkey anti-mouse A21202, Invitrogen, 1:200]. Sections containing DR were stained for tryptophan hydroxylase 2 (TPH2, primary antibody: rat anti-TPH2 ab111828, Abcam, 1:600; secondary antibody: Alexa 555 donkey anti-rabbit, Invitrogen, 1:200) and NeuN. Sections containing LC and DR axons in M1 were stained for norepinephrine transporter (NET, primary antibody: mouse IgG1 anti-NET MA5-24647, ThermoFisher, 1:1000; secondary antibody: Alexa 488 donkey anti-mouse, Invitrogen, 1:200) and serotonin transporter (SERT, primary antibody: recombinant anti-SERT antibody, ab254358, 1:2000; secondary antibody, Alexa 555 donkey anti-rabbit, Invitrogen, 1:200). Sections were mounted with ProLong Diamond Antifade Mountant (Invitrogen) and coverslipped on glass slides (Electron Microscopy Sciences).

### Stereological analysis

Sections were examined on a Zeiss microscope with a motorized stage and a digital camera controlled by Stereo Investigator software (version 2020.2.2, MBF Bioscience). Anatomical boundaries were traced under 2.5X/0.085NA objective lens and counting was performed under a 63X/1.4NA lens. TH^+^ cells were counted throughout the LC and TPH2^+^ cells throughout the DR using the optical fractionator probe (West, 2012). A 150 μm × 150 μm counting frame and grid size of 275 μm × 175 μm for LC and 250 μm × 175 μm for DR was used with a 2 μm guard zone for both the top and bottom. The coefficient of error Gundersen (*m* = 1), an indicator of counting precision (Gundersen et al., 1999), ranged from 0.04 to 0.09 (LC) and 0.03 to 0.04 (DR). For regions that showed meth-induced degeneration, NeuN^+^ cells were counted using the above parameters to confirm cell loss and not mere phenotypic suppression. NET^+^ and SERT^+^ axon length in M1 was quantified using the Spaceballs probe (Mouton et al., 2002; West, 2018), using a hemisphere of 20 μm radius, a grid size of 250 μm × 250 μm, and a guard zone of 3 μm, resulting in a Gundersen coefficient or error (*m* = 1) of 0.03 to 0.06 (NET^+^ axon) and 0.02 to 0.05 (SERT^+^ axon).

### Statistical analyses

Data were analyzed in Prism (GraphPad Software, La Jolla, CA, United States); normality was tested using the Shapiro-Wilk test. One-way ANOVA with Tukey *post-hoc* or unpaired Student *t*-test were used for datasets that passed normality whereas the non-parametric Kruskal-Wallis with Dunn’s *post-hoc* or Mann-Whitney tests were used for datasets failing normality testing. Data are presented as individual plots with mean and standard error of the mean. Statistical details pertaining to each experiment are provided in figure legends; α = 0.05.

## Results

### Meth increased monoamine oxidase-dependent mitochondrial oxidant stress in locus coeruleus and dorsal raphe axons

**FIGURE 1:**
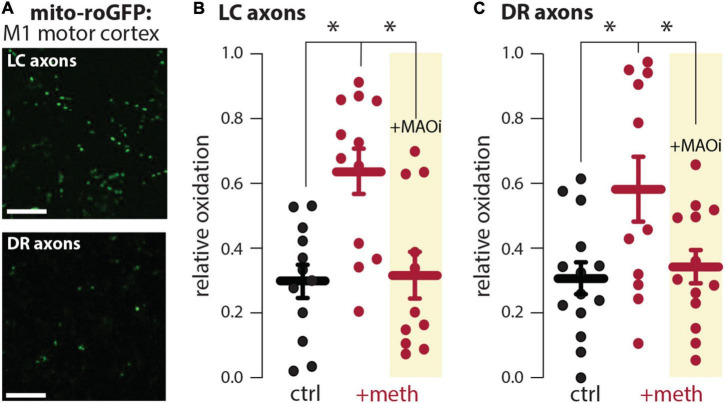
Meth increased monoamine oxidase (MAO)-dependent mitochondrial oxidant stress in locus coeruleus (LC) and dorsal raphe (DR) axons. **(A)** Sample image (top) in the primary motor cortex showing expression of the redox biosensor roGFP targeted to the mitochondrial matrix (mito-roGFP) in LC axons; scale bar denotes 10 μm. Sample image (bottom) in the primary motor cortex showing the redox biosensor mito-roGFP expressed in DR axons; scale bar denotes 10 μm. **(B)** Compared to aCSF perfusion (ctrl), meth (+meth, 10 μM) increased LC axonal mitochondrial oxidant stress which was attenuated by the MAO inhibitor rasagiline (+MAOi, 1 μM; ctrl *n* = 12 brain slices/4 mice, +meth *n* = 12 brain slices/4 mice, +MAOi *n* = 11 brain slices/4 mice). Data analyzed using Kruskal-Wallis [*H*_(2)_ = 11.60, *p* = 0.0030] with Dunn’s *post-hoc* (ctrl vs. +meth, *p* = 0.0090; ctrl vs. +MAOi, *p* > 0.9999; +meth vs. +MAOi, *p* = 0.0109). **(C)** +meth similarly increased mitochondrial oxidant stress in DR axons in the primary motor cortex and +MAOi attenuated the increased mitochondrial oxidant stress; ctrl *n* = 14 brain slices/5 mice, +meth *n* = 11 brain slices/3 mice, +MAOi *n* = 13 brain slices/6 mice. Data analyzed using one-way ANOVA [*F*_(2,35)_ = 4.746, *p* = 0.0150] with Tukey *post-hoc* (control vs. +meth, *p* = 0.0173; control vs. +MAOi, *p* = 0.9151; +meth vs. +MAOi, *p* = 0.0474); *p < 0.05.

**FIGURE 2:**
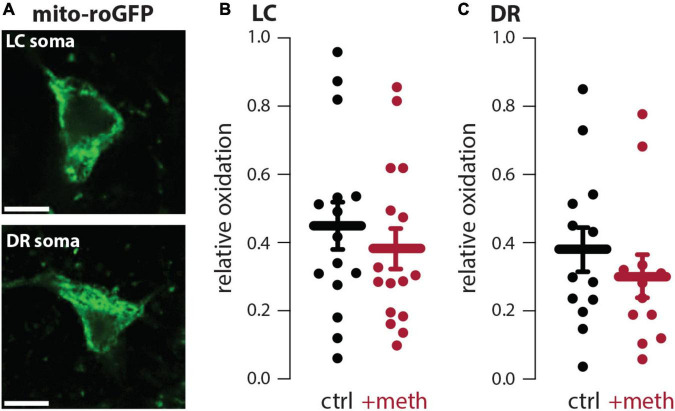
Meth did not increase somatic mitochondrial oxidant stress in locus coeruleus (LC) or dorsal raphe (DR) neurons. **(A)** Sample image (top) showing mito-roGFP expressed in the soma of a LC norepinephrine neuron; scale bar denotes 10 μm. Sample image (bottom) showing mito-roGFP expressed in the soma of a DR serotonin neuron; scale bar denotes 10 μm. **(B)** Meth (+meth; 10 μM) had no effect on somatic mitochondrial oxidant stress in LC norepinephrine neurons; ctrl *n* = 15 neurons/7 mice, + meth *n* = 16 neurons/4 mice. Data analyzed using an unpaired *t*-test [*t*_(29)_ = 0.7229, *p* = 0.4755, two-tailed]. **(C)** +meth also had no effect on somatic mitochondrial oxidant stress in DR serotonin neurons (ctrl *n* = 13 neurons/4 mice, +meth *n* = 12 neurons/4 mice). Data analyzed using Mann-Whitney test (*U* = 61, *p* = 0.3760, two-tailed).

In dopamine neurons meth disrupts VMAT2 function resulting in elevated levels of cytosolic dopamine (Sulzer et al., 2005; Freyberg et al., 2016); this elevated cytosolic dopamine gets metabolized by MAO enzymes resulting in mitochondrial oxidant stress in axonal compartments of SNc and VTA dopamine neurons (Graves et al., 2020; Du et al., 2021). VMAT2 is not exclusive to midbrain dopamine neurons and similarly packages norepinephrine and serotonin into vesicles. Furthermore, MAO enzymes metabolize not only dopamine, but also norepinephrine and serotonin. Meth was therefore hypothesized to increase mitochondrial oxidant stress in LC and DR axons due to MAO metabolism. To test this hypothesis, LC and DR axonal mitochondrial oxidant stress was measured in the primary motor cortex. Bath application of meth (10 μM) increased mitochondrial oxidant stress in both LC and DR axons and this meth-induced stress was attenuated by MAO inhibition (1 μM rasagiline) (Figure 1), consistent with what is seen in both VTA and SNc axons (Graves et al., 2020; Du et al., 2021). A notable feature of meth-induced MAO-dependent mitochondrial oxidant stress is that it is apparent in axonal but not somatic compartments in SNc and VTA dopamine neurons (Graves et al., 2020; Du et al., 2021). To determine whether this compartmentalization held true for noradrenergic and serotonergic neurons we measured the effect of meth on mitochondrial oxidant stress in the somatic compartment of LC and DR neurons; meth had no effect on somatic mitochondrial oxidant stress in LC norepinephrine or DR serotonin neurons (Figure 2).

### Locus coeruleus neurons were vulnerable and dorsal raphe neurons were resistant to chronic meth-induced degeneration

**FIGURE 3:**
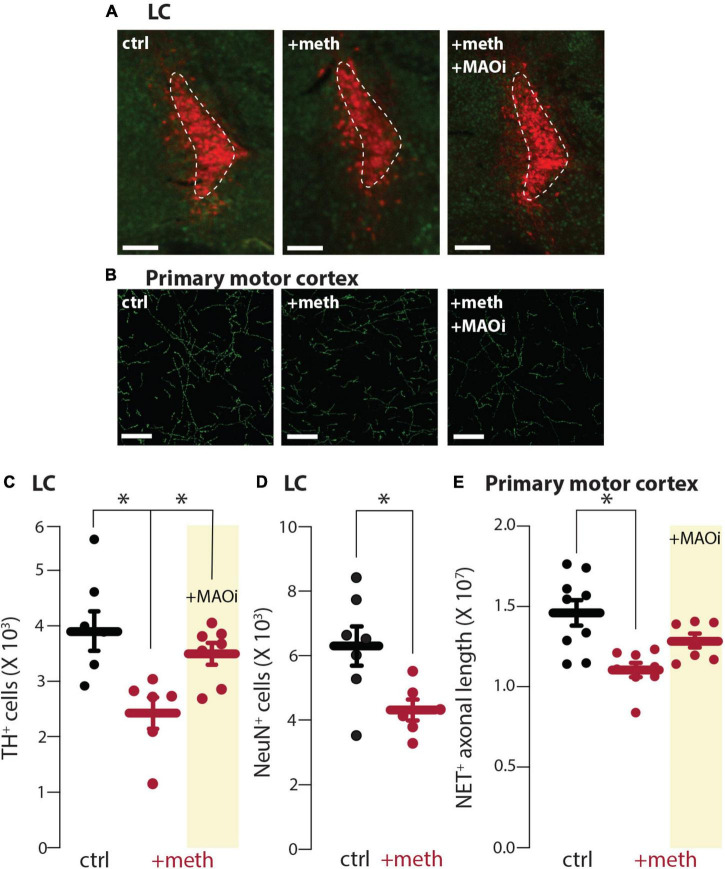
Chronic meth administration induced locus coeruleus (LC) degeneration and monoamine oxidase (MAO) inhibition was neuroprotective. **(A)** Sample images depicting loss of tyrosine hydroxylase expressing (TH^+^) neurons in LC of meth (+meth; 5 mg/kg)-compared to saline (ctrl; 10 ml/kg)-treated mice and neuroprotection by pretreatment of the MAO inhibitor rasagiline (+MAOi; 1 mg/kg); scale bars denote 200 μm; TH stained in red with NeuN counterstained in green. **(B)** Sample images depicting NET^+^ axons in the primary motor cortex of ctrl, +meth, and +MAOi mice; scale bars denote 20 μm. **(C)** Quantified data indicate that chronic meth induced loss of TH^+^ cells in the LC of meth-treated mice and this loss was prevented by pretreatment of rasagiline; ctrl *n* = 7, +meth *n* = 6, +MAOi *n* = 7 mice. Data analyzed using one-way ANOVA [*F*_(2,17)_ = 6.585, *p* = 0.0076] with Tukey *post-hoc* (ctrl vs. +meth, *p* = 0.0067; ctrl vs. +MAOi, *p* = 0.5891; +meth vs. +MAOi, *p* = 0.0484). **(D)** Quantified data indicate that chronic meth induced loss of NeuN^+^ cells in the LC of meth-treated mice; ctrl *n* = 7, +meth *n* = 6. Data analyzed using an unpaired *t*-test [*t*_(11)_ = 2.746, *p* = 0.0190, two-tailed]. **(E)** Quantified data show loss of NET^+^ axons in +meth mice; ctrl *n* = 9, +meth *n* = 8, +MAOi *n* = 7 mice. Data analyzed using one-way ANOVA [*F*_(2,21)_ = 8.670, *p* = 0.0018] with Tukey *post-hoc* (ctrl vs. +meth, *p* = 0.0012; ctrl vs. +MAOi, *p* = 0.1360; +meth vs. +MAOi, *p* = 0.1479); *p < 0.05.

Chronic meth (5 mg/kg, i.p. for 28 days) leads to SNc degeneration which is prevented by MAO inhibition (Du et al., 2021; Graves et al., 2021). In contrast, the VTA is resistant to degeneration despite having similar meth-induced MAO-dependent mitochondrial oxidant stress in axons (Du et al., 2021). To determine whether LC and DR neurons were vulnerable or resistant to chronic meth-induced degeneration mice were treated for 28 consecutive days with 5 mg/kg meth and the number of LC and DR neurons stereologically counted. The number of TH^+^ neurons in the LC and LC axon length measured in the primary motor cortex was significantly decreased in mice treated with meth compared to saline-treated controls (Figures 3A–C,E). To confirm this loss was not resultant from phenotypic suppression, sections were counterstained for NeuN, a non-specific neuronal marker; the number of NeuN^+^ cells in the LC were also decreased in meth treated subjects (Figure 3D), indicating that chronic meth resulted in neurodegeneration, not phenotypic suppression. To determine whether degeneration was MAO-dependent, as is the case for chronic meth-induced SNc degeneration (Du et al., 2021; Graves et al., 2021), a separate group of mice was administered the MAO inhibitor rasagiline (1 mg/kg) as a 30-min pretreatment prior to each meth injection. Consistent with prior results in the SNc (Du et al., 2021; Graves et al., 2021), MAO inhibition prevented LC degeneration (Figures 3C,E).

**FIGURE 4:**
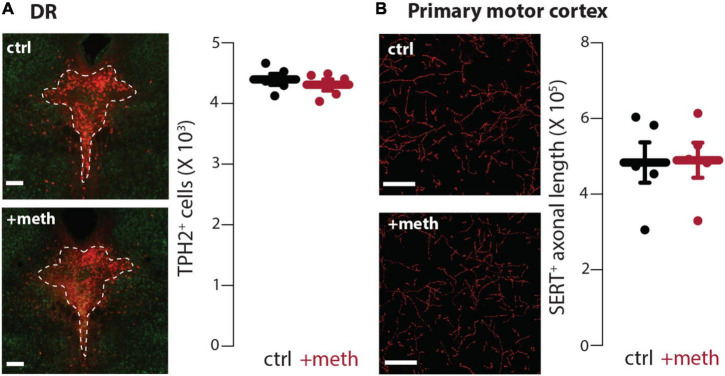
Dorsal raphe (DR) neurons were resistant to chronic meth-induced degeneration. **(A)** Sample images (left) depicting tryptophan hydroxylase expressing (TPH2^+^) neurons in DR of saline (ctrl; 10 ml/kg)-and meth (+meth; 5 mg/kg)-treated mice; scale bars denote 200 μm; TPH stained in red with NeuN counterstained in green. Quantified data (right) indicate that chronic meth had no effect on number of TPH2^+^ cells in the DR; ctrl *n* = 5 and +meth *n* = 5 mice. Data analyzed using an unpaired *t*-test [*t*_(8)_ = 0.6708, *p* = 0.5212, two-tailed]. **(B)** Sample images (left) depicting SERT^+^ axons in the primary motor cortex of ctrl and +meth mice; scale bars denote 20 μm. Quantified data (right) show no difference in axon length between ctrl and +meth mice; ctrl *n* = 5 and +meth *n* = 5 mice. Data analyzed using an unpaired *t*-test [*t*_(8)_ = 0.07813, *p* = 0.9396, two-tailed].

In contrast to the LC, DR neurons were resistant to chronic meth-induced degeneration (Figure 4A) despite having similar meth-induced MAO-dependent axonal mitochondrial oxidant stress as LC neurons (Figure 1). Because meth increased axonal but not somatic mitochondrial oxidant stress, we also stereologically quantified the length of SERT^+^ axons in the primary motor cortex to assess whether chronic meth was detrimental to serotonergic axons. Meth, however, did not affect the length of SERT^+^ axons (Figure 4B). These results mirror those seen in midbrain dopamine neurons wherein chronic meth administration produced axonal and somatic degeneration of SNc but not VTA dopamine neurons (Du et al., 2021).

### L-type Ca^2+^ channel activity increased axonal mitochondrial oxidant stress in locus coeruleus but not dorsal raphe axons

**FIGURE 5:**
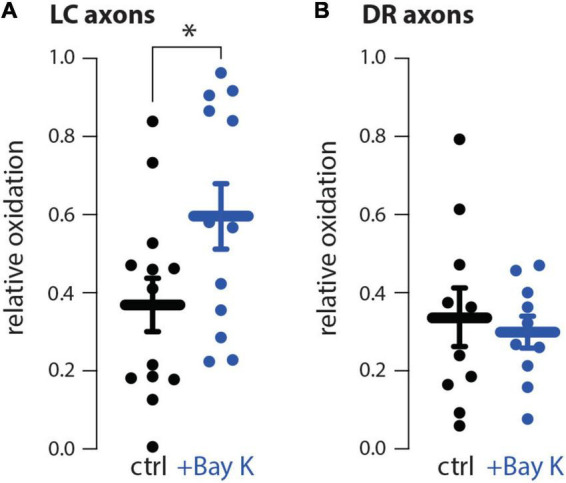
The L-type Ca^2+^ channel activator Bay K8644 increased mitochondrial oxidant stress in locus coeruleus (LC) but not dorsal raphe (DR) axons. **(A)** Compared to aCSF perfusion (ctrl), L-type Ca^2+^ channel activation using 10 μM Bay K8644 (+Bay K) increased mitochondrial oxidant stress in LC axons; ctrl *n* = 13 brain slices/4 mice, +Bay K *n* = 12 brain slices/4 mice. Data analyzed using an unpaired *t*-test [*t*_(23)_ = 2.119, *p* = 0.0451, two-tailed]. **(B)** +Bay K did not increase DR axonal mitochondrial oxidant stress; ctrl *n* = 10 brain slices/4 mice, +Bay K *n* = 10 brain slices/4 mice. Data analyzed by unpaired *t*-test [*t*_(18)_ = 2.119, *p* = 0.4312, two-tailed]; *p < 0.05.

In our prior study, the differential vulnerability of SNc and VTA neurons was attributed to the presence of L-type Ca^2+^ channel-dependent mitochondrial oxidant stress (Du et al., 2021). SNc neurons, which are vulnerable to meth-induced degeneration, have L-type Ca^2+^ channel-dependent mitochondrial oxidant stress in both somatic and axonal compartments, whereas, VTA neurons, which are resistant to meth-induced degeneration, lack L-type Ca^2+^ channel-dependent mitochondrial oxidant stress in both somatic and axonal compartments (Guzman et al., 2010; Graves et al., 2020, 2021; Du et al., 2021). We hypothesized a similar dichotomy differentiating LC and DR neurons. To test this hypothesis, L-type Ca^2+^ channel activity was evoked in axons by bath application of Bay K8644 (10 μM), an L-type Ca^2+^ channel activator. Activating L-type Ca^2+^ channel activity increased mitochondrial oxidant stress in LC but not DR axons (Figure 5).

### L-type Ca^2+^ channel inhibition was neuroprotective

**FIGURE 6:**
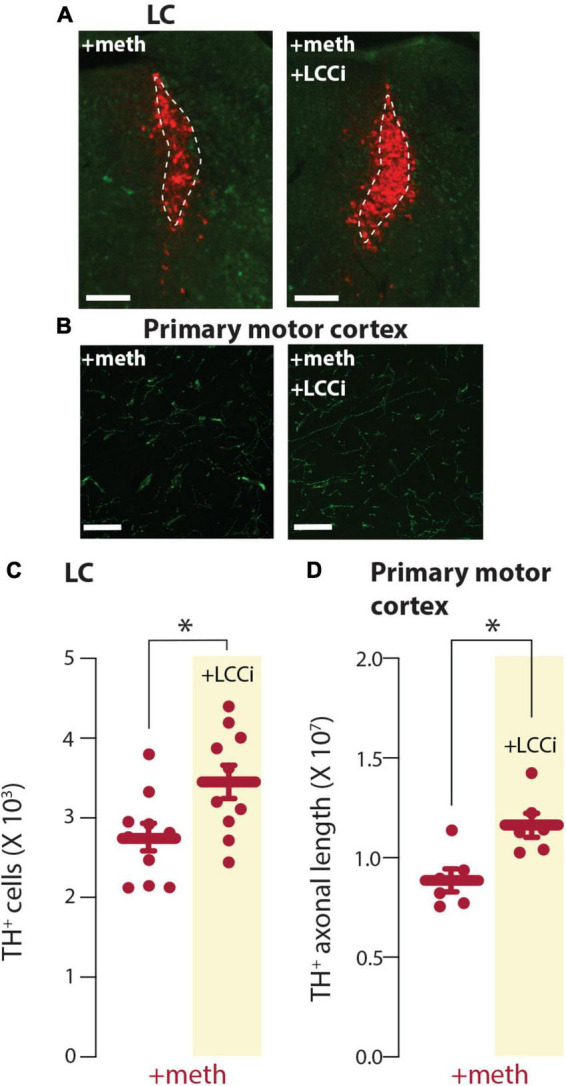
L-type Ca^2+^ channel inhibition prevented chronic meth-induced degeneration of locus coeruleus (LC) neurons. **(A)** Sample images depicting neuroprotection by the L-type Ca^2+^ channel inhibitor isradipine (+LCCi; 3 mg/kg/day) to chronic meth (+meth; 5 mg/kg)-induced LC neuron loss; scale bars denote 200 μm; TH stained in red with NeuN counterstained in green. **(B)** Sample images depicting neuroprotection by the L-type Ca^2+^ channel inhibitor isradipine (+LCCi; 3 mg/kg/day) to chronic meth (+meth; 5 mg/kg)-induced LC axon loss; axons in the primary motor cortex were stained for NET; scale bars 20 μm. **(C)** Stereological quantification of TH^+^ cells indicated the loss of TH^+^ cells in LC of chronic meth-treated mice was prevented by +LCCi; +meth *n* = 10 and +LCCi *n* = 10 mice. Data analyzed using an unpaired *t*-test [*t*_(18)_ = 2.601, *p* = 0.0181, two-tailed]. **(D)** Stereological quantification of NET^+^ axon length indicated the loss of NET^+^ axons in the primary motor cortex of chronic meth treated mice was prevented by +LCCi; +meth *n* = 6 and +LCCi *n* = 6 mice. Data analyzed using an unpaired *t*-test [*t*_(10)_ = 3.334, *p* = 0.0076, two-tailed]; *p < 0.05.

L-type Ca^2+^ channel inhibition prevented chronic meth-induced SNc degeneration supporting the hypothesis that L-type Ca^2+^ channel-dependent mitochondrial oxidant stress differentiates vulnerable vs. resistant neurons. To determine if this is the case for LC neurons the L-type Ca^2+^ channel inhibitor isradipine (3 mg/kg/day) or vehicle was delivered *via* osmotic minipumps and mice were treated with meth (5 mg/kg) for 28 days. L-type Ca^2+^ channel inhibition attenuated chronic meth-induced degeneration at both the somatic and axonal level (Figure 6), indicating that L-type Ca^2+^ channel activity is necessary for chronic meth-induced LC degeneration.

## Discussion

The present study investigated the differential vulnerability of noradrenergic LC and serotonergic DR neurons to chronic meth-induced degeneration. Findings from the LC and DR mirror those recently reported for SNc and VTA dopamine neurons (Du et al., 2021). Like in SNc dopamine neurons, which are vulnerable to chronic meth-induced degeneration, meth increased MAO-dependent mitochondrial oxidant stress in LC axons and MAO inhibition prevented meth-induced neurodegeneration. In contrast, DR serotonergic neurons, like VTA dopamine neurons, had meth-induced MAO-dependent mitochondrial oxidant stress in axons but were resistant to degeneration. Together these studies indicate that meth-induced MAO-dependent stress is necessary and is a driver of degeneration; however, it is not sufficient for degeneration. A second source of mitochondrial stress in SNc (Guzman et al., 2010; Du et al., 2021) and LC neurons is L-type Ca^2+^ channel-dependent mitochondrial stress, whereas L-type Ca^2+^ channel-dependent stress is not detected in VTA (Guzman et al., 2010; Du et al., 2021) or DR neurons, which were resistant to degeneration. Given that the L-type Ca^2+^ channel negative allosteric modulator isradipine was neuroprotective, this suggests that the combination of L-type Ca^2+^ channel- and meth-induced MAO-dependent mitochondrial stress is required for degeneration. These findings support the hypothesis that L-type Ca^2+^ channel- and meth-induced MAO-dependent mitochondrial stress converge in LC and SNc axons with degeneration being driven by MAO metabolism of cytosolic neurotransmitter and vulnerability determined by L-type Ca^2+^-dependent stress. Meth-induced dysfunction of VMAT2 results in elevated levels of cytosolic monoamines which have been thought, particularly in reference to dopamine, to have deleterious effects. The reason for this is cytosolic dopamine can readily auto-oxidize to form reactive quinones which would generate cytosolic oxidant stress (Sulzer and Zecca, 2000). Additionally, cytosolic dopamine could be metabolized by MAO enzymes which were thought to generate hydrogen peroxide from electrons released into the cytosol by the deamination process, also thought to increase cytosolic oxidant stress (Edmondson et al., 2009; Edmondson, 2014). However, further investigation reveals that cytosolic dopamine is metabolized by MAO enzymes, but the electrons generated are transferred to the mitochondrial intermembrane space, not the cytosol; this provides bioenergetic support but also produces mitochondrial oxidant stress (Graves et al., 2020). Norepinephrine and serotonin, like dopamine, can also auto-oxidize (Louis et al., 1992; Wrona and Dryhurst, 1998; Manini et al., 2007); however, in the current study meth increased MAO-dependent mitochondrial oxidant stress arguing that this MAO-dependent electron transfer is likely a shared mechanism spanning monoamine systems.

While a built-in mechanism to provide on-demand bioenergetic support would seemingly be beneficial, disrupting VMAT2 function *via* meth administration to cause vesicular dumping of neurotransmitter into the cytosol is in excess of what would be presumably experienced by normal turnover during reuptake, packaging, and release. Persistently disrupting VMAT2 function for a protracted period with 28 daily injections of meth produces axonal and somatic degeneration of LC but not DR neurons, analogous to the effects in SNc and VTA neurons, respectively (Du et al., 2021; Graves et al., 2021). The decreased number of LC neurons likely indicates overt and irreversible neuronal loss given that these are post-mitotic, non-dividing neurons and the number of cells in the LC stained for NeuN, which is the neuron-specific nuclear splicing regulator Fox 3 (Kim et al., 2009), was also decreased. Nevertheless, future studies using additional methods to identify degeneration would be of value to definitively confirm irreversible neuronal loss. For example, silver staining has been used to identify degeneration of substantia nigra pars compacta dopamine neurons after an acute meth binge in mice (Ares-Santos et al., 2014). A limited number of studies have investigated the deleterious effects of meth on noradrenergic neurons. Using binge paradigms, meth generally does not result in NE depletion (Wagner et al., 1979; Wagner and Davies, 1980; Ricaurte et al., 1984; Commins and Seiden, 1986). However, administration of either 5 or 15 mg/kg meth every 12 h for four consecutive days results in decreased norepinephrine uptake sites with the 15 mg/kg dose also decreasing levels of norepinephrine in rats (Zaczek et al., 1989). In non-human primates 3–6 months administration of intravenous meth (eight injections/day) produces a persistent decrease in levels of norepinephrine (Seiden et al., 1976). Transgenic mice with reduced expression of VMAT2 show progressive LC (Taylor et al., 2014) and SNc degeneration (Caudle et al., 2007) but not serotonergic degeneration (Alter et al., 2016) similar to the effects of chronic meth on monoaminergic degeneration (Du et al., 2021; Graves et al., 2021). Furthermore, mice with decreased VMAT2 expression are more sensitive to the neurotoxic effects of meth when administered in an acute binge paradigm and increasing VMAT2 attenuates the deleterious effects of an acute meth binge (Fumagalli et al., 1999; Guillot et al., 2008a,b; Lohr et al., 2015).

Although chronic meth administration did not result in serotonergic degeneration, studies using an acute binge model of meth administration produce serotonin depletion (Hotchkiss and Gibb, 1980; Peat et al., 1985; Commins and Seiden, 1986; Ricaurte et al., 1989; Schmidt et al., 1991; Green et al., 1992; Pu and Vorhees, 1995) and decreased activity of SERT (Haughey et al., 2000; Sekine et al., 2006; Kish et al., 2010; Huang et al., 2019) and TPH (Hotchkiss and Gibb, 1980), whereas, 32 mg/kg/day for 5 days produces a decrease in 5-HT uptake sites (Armstrong and Noguchi, 2004); for review see Krasnova and Cadet (2009). These results are not inherently in conflict with our findings as tissue content of neurotransmitter, protein expression, and/or protein function are not necessarily indicative of degeneration or degenerative processes. In the current report we determined axon length and number of neurons using stereological methods and did not examine relative levels of protein expression or function. Further study will be necessary to determine whether neuronal populations such as the DR and VTA exhibit functional deficits in response to chronic meth. Consistent with this, mice with reduced VMAT2 expression do not develop serotonergic degeneration but do show diminished serotonin release (Alter et al., 2016).

All monoaminergic neuronal populations in the current study (LC and DR) and recent report (SNc and VTA) have similar meth-induced MAO-dependent mitochondrial oxidant stress but only SNc and LC neurons were vulnerable to degeneration (Du et al., 2021; Graves et al., 2021). One differentiating factor distinguishing vulnerable from resistant neurons is the presence of mitochondrial oxidant stress resulting from L-type Ca^2+^ channel activity (Chan et al., 2007; Guzman et al., 2010, 2018; Sanchez-Padilla et al., 2014; Du et al., 2021). Besides MAO-dependent mitochondrial stress, another source of mitochondrial stress is from calcium that enters the cell through L-type Ca^2+^ channels. L-type Ca^2+^ channel-dependent mitochondrial stress is present in axonal and somatic compartments of SNc but not VTA dopamine neurons (Guzman et al., 2010; Du et al., 2021). Similar to SNc dopamine neurons, LC norepinephrine neurons also exhibit L-type Ca^2+^ channel-dependent mitochondrial oxidant stress in the soma (Sanchez-Padilla et al., 2014). Consistent with somatic presence, the present study showed L-type Ca^2+^ channel-dependent mitochondrial oxidant stress in LC axons. In both SNc and LC neurons L-type Ca^2+^ channel-dependent mitochondrial oxidant stress is primarily attributed to calcium oscillations mediated by Cav_1.3_ channels (Chan et al., 2007; Guzman et al., 2010, 2018; Sanchez-Padilla et al., 2014). Current thinking is that calcium influx increases SNc mitochondrial oxidant stress due to poor intrinsic buffering capacity (Foehring et al., 2009; Guzman et al., 2010; Zampese and Surmeier, 2020). LC neurons similarly have poor calcium buffering capacity (Sanchez-Padilla et al., 2014) and limited expression of calcium buffering proteins (Lamerand et al., 2020). In contrast, VTA neurons, which express Cav_1.3_ L-type Ca^2+^ channels (Sukiasyan et al., 2009), also express higher levels of calbindin, a calcium buffering protein, than SNc neurons (Liang et al., 1996), which is likely protecting mitochondria from elevated intracellular calcium concentrations. DR neurons also express Cav_1.3_ channels (Sukiasyan et al., 2009) and there is evidence of calbindin expression within the raphe (Iacopino and Christakos, 1990; Sequier et al., 1990). Given that L-type Ca^2+^ channel activation using Bay K did not increase mitochondrial oxidant stress in DR axons it stands to reason that DR neurons, similar to VTA dopamine neurons, likely express sufficient levels of calcium buffering proteins, such as calbindin, to minimize mitochondrial stress resultant from calcium influx.

It is important to note that only male subjects were used in the current study and in our recent report (Du et al., 2021) detailing the differential vulnerability of SNc and VTA neurons to chronic meth-induced degeneration. The reason our previous and current investigations were limited to male subjects is that female subjects are less vulnerable to the nigrostriatal damage elicited by an acute binge of meth (Dluzen and McDermott, 2000; Yu and Liao, 2000; Gajjar et al., 2003; Liu and Dluzen, 2006; Bourque et al., 2011b). Based on the existing evidence and seemingly neuroprotective effect of estrogen (Dluzen and McDermott, 2006; Bourque et al., 2011a), we hypothesize that female subjects would also be more resistant to chronic meth-induced degeneration of SNc neurons. Given that chronic meth similarly induces LC degeneration, seemingly *via* the same mechanisms, i.e., the presence of both L-type Ca^2+^ channel- and meth-induced MAO-dependent mitochondrial oxidant stress, it is likely the case that LC neurons would also be resistant in female subjects. Nonetheless, future studies addressing sex differences relating to chronic meth-induced degeneration of monoaminergic neurons are needed. A second factor to consider is that subjects that underwent chronic administration of saline, meth, or rasagiline + meth were sacrificed within 12 h of the last injection. In mice, an acute binge of meth results in axon and cell body loss of SNc dopamine neurons within 3–12 h after the last meth administration and, for axons, peaks after 1 day (Ares-Santos et al., 2014; Granado et al., 2018). This peak in axonal loss is followed by partial recovery during protracted abstinence whereas somatic degeneration remains stable (Ares-Santos et al., 2014; Granado et al., 2018). The time course of axonal and somatic loss during the chronic administration period in the current study is unclear. Additionally, it is unclear whether there is recovery during abstinence after chronic meth. However, in rats trained to self-administer meth for 3 h per day for 14 consecutive days, dopaminergic axonal loss in the dorsal striatum did not manifest until 14 days of abstinence (Kousik et al., 2014). In contrast to the partial recovery seen with an acute binge (Ares-Santos et al., 2014; Granado et al., 2018), more protracted abstinence (up to 56 days) from meth self-administration resulted in a more pronounced loss of axons, not recovery suggesting progressive degeneration (Kousik et al., 2014) during abstinence from chronic daily exposure to meth.

Results from the current and recent study examining differential vulnerability of SNc and VTA neurons (Du et al., 2021) parallels the differential vulnerability of neuronal populations in Parkinson’s disease (Gonzalez-Rodriguez et al., 2020). The presence of L-type Ca^2+^ channel-dependent mitochondrial oxidant stress and poor calcium buffering capacity is one mechanism thought to underly vulnerability to degeneration in Parkinson’s disease. The SNc, LC, and dorsal motor nucleus of the vagus all have Cav_1.3_ L-type Ca^2+^ oscillations that contribute to mitochondrial oxidant stress, poor calcium buffering capacity, and are neuronal populations particularly vulnerable to degeneration in Parkinson’s disease (Guzman et al., 2009, 2010, 2018; Goldberg et al., 2012; Sanchez-Padilla et al., 2014; Surmeier et al., 2017; Gonzalez-Rodriguez et al., 2020; Zampese and Surmeier, 2020). Consistent with the parallels between chronic meth-induced degeneration of SNc and LC neurons, meth increases the risk for developing Parkinson’s disease (Callaghan et al., 2010, 2012; Granado et al., 2013; Curtin et al., 2015; Moratalla et al., 2017; Lappin and Darke, 2021; Salari et al., 2022) and even increases synuclein expression in the SNc and gut of rats trained to self-administer meth (Persons et al., 2021), although see Moszczynska et al. (2004) and Kish et al. (2017). Given the similarities between Parkinson’s disease and meth neurotoxicity/neurodegeneration, meth administration has potential value as a preclinical model of Parkinson’s disease (Shin et al., 2021). Whether chronic meth-induced degeneration of SNc and LC neurons occur independently or are linked remains unclear. Lesioning LC neurons increase the sensitivity of SNc neurons to MPTP-induced degeneration in non-human primates (Mavridis et al., 1991) and mice (Bing et al., 1994; Yao et al., 2015) whereas chemogenetic enhancement of LC neuronal activity is neuroprotective in the A53T mouse model of Parkinson’s disease (Jovanovic et al., 2022). Similarly, lesioning the LC enhances dopamine depletion resultant from an acute binge of meth (5 mg/kg × 2, 2 h interval) (Fornai et al., 1995). Additional study is necessary to ascertain whether LC and SNc degeneration are linked in the context of chronic meth-induced neurodegeneration. Furthermore, the question remains as to whether degeneration continues to progress throughout abstinence and if so whether LC loss shapes the trajectory of SNc degeneration.

In rats trained to self-administer meth using a 14-day short access paradigm, loss of TH^+^ SNc neurons is not observed until 28 days of abstinence (Kousik et al., 2014). Similarly, in mice administered 5 mg/kg meth for 14 days, SNc degeneration did not become apparent until 14 days of abstinence (Graves et al., 2021). During abstinence SNc neurons had increased axonal and somatic mitochondrial oxidant stress sensitive to MAO and L-type Ca^2+^ channel inhibition, respectively (Graves et al., 2021). This increased MAO and L-type Ca^2+^ sensitive mitochondrial oxidant stress was associated with decreased VMAT2 mRNA and accelerated pacemaking frequency (Graves et al., 2021). An acute meth binge also decreases VMAT2 in rodents and is due to VMAT2 nitrosylation (Eyerman and Yamamoto, 2005, 2007). Decreased VMAT2 during abstinence from meth would presumably result in elevated levels of cytosolic neurotransmitter due to a diminished ability to adequately package neurotransmitter into vesicles thereby resulting in a persistent MAO-dependent mitochondrial stress in axons, whereas accelerated pacemaking would increase the bioenergetic demand and overall calcium burden experienced by the cell thereby contributing to mitochondrial stress. One potential mechanism underlying accelerated pacemaking is oxidant stress-mediated impairment of A-type Kv4.3 potassium channels (Subramaniam et al., 2014). It is likely that LC neurons are similarly impacted, mirroring SNc neurons, and would continue to degenerate during abstinence.

The present study demonstrated that MAO inhibition prevented LC from chronic meth-induced degeneration, indicating a necessary role of MAO-dependent mitochondrial stress. But given that DR does not degenerate despite having the same MAO-dependent mitochondrial stress, it is not sufficient to drive chronic meth-induced degeneration. L-type Ca^2+^ channel inhibition by isradipine also provided neuroprotection against chronic meth-induced LC degeneration, suggesting that both MAO-and L-type Ca^2+^ channel-dependent mitochondrial stress are required for meth-induced degeneration. A question remains as to whether degeneration progresses during abstinence and if so whether MAO- and L-type Ca^2+^ channel-dependent stress continue to contribute; existing evidence suggests this is the case for SNc neurons (Kousik et al., 2014; Graves et al., 2021) and we would expect similar outcomes in LC neurons. Although VTA neurons are resistant to chronic meth-induced degeneration (Du et al., 2021) they do not seem to be impervious. In rats trained to self-administer meth VTA neurons do eventually degenerate after 56 days of abstinence whereas SNc loss is evident after 28 days of abstinence (Kousik et al., 2014). Whether serotonergic neurons are similarly lost during protracted abstinence from meth and potential underlying mechanisms are unclear and in need of further study.

Lastly, current findings emphasize the need for dual purpose pharmacotherapies to treat patients suffering from a meth use disorder. Not only is there a need to develop treatment strategies to maintain abstinence but treatment strategies will also be needed to provide neuroprotection and mitigate the risk for developing neurodegenerative diseases such as Parkinson’s disease. It is unclear whether MAO inhibition might ameliorate maladaptive behaviors such as meth-seeking; however, L-type Ca^2+^ channel inhibition with isradipine attenuates acquisition and facilitates extinction (also blocking subsequent reinstatement) of cocaine-induced conditioned place preference (Degoulet et al., 2016), and attenuates cue-associated seeking behavior in rats trained to self-administer cocaine (Addy et al., 2018), suggesting that isradipine may have translational value with the potential to support abstinence and provide neuroprotection in patients with a meth use disorder.

## Data availability statement

The raw data supporting the conclusions of this article will be made available by the authors, without undue reservation.

## Ethics statement

The animal study was reviewed and approved by University of Minnesota Institutional Animal Care and Use Committee.

## Author contributions

SMG and YD designed the experiments and drafted the manuscript. YD, SC, and AP conducted the experiments and analyzed the data. All authors reviewed the manuscript and approved the submitted version.

## Conflict of interest

The authors declare that the research was conducted in the absence of any commercial or financial relationships that could be construed as a potential conflict of interest.

## Publisher’s note

All claims expressed in this article are solely those of the authors and do not necessarily represent those of their affiliated organizations, or those of the publisher, the editors and the reviewers. Any product that may be evaluated in this article, or claim that may be made by its manufacturer, is not guaranteed or endorsed by the publisher.
